# Understanding Multilevel Selection May Facilitate Management of Arbuscular Mycorrhizae in Sustainable Agroecosystems

**DOI:** 10.3389/fpls.2020.627345

**Published:** 2021-01-26

**Authors:** Nancy Collins Johnson, Kara Skye Gibson

**Affiliations:** ^1^ School of Earth & Sustainability, Northern Arizona University, Flagstaff, AZ, United States; ^2^ Department of Biological Sciences, Northern Arizona University, Flagstaff, AZ, United States

**Keywords:** complex adaptive systems, emergent properties, local adaptation, mycorrhizal phenotype, arbuscular mycorrhizae, high-input agriculture

## Abstract

Studies in natural ecosystems show that adaptation of arbuscular mycorrhizal (AM) fungi and other microbial plant symbionts to local environmental conditions can help ameliorate stress and optimize plant fitness. This local adaptation arises from the process of multilevel selection, which is the simultaneous selection of a hierarchy of groups. Studies of multilevel selection in natural ecosystems may inform the creation of sustainable agroecosystems through developing strategies to effectively manage crop microbiomes including AM symbioses. Field experiments show that the species composition of AM fungal communities varies across environmental gradients, and that the biomass of AM fungi and their benefits for plants generally diminish when fertilization and irrigation eliminate nutrient and water limitations. Furthermore, pathogen protection by mycorrhizas is only important in environments prone to plant damage due to pathogens. Consequently, certain agricultural practices may inadvertently select for less beneficial root symbioses because the conventional agricultural practices of fertilization, irrigation, and use of pesticides can make these symbioses superfluous for optimizing crop performance. The purpose of this paper is to examine how multilevel selection influences the flow of matter, energy, and genetic information through mycorrhizal microbiomes in natural and agricultural ecosystems, and propose testable hypotheses about how mycorrhizae may be actively managed to increase agricultural sustainability.

## Introduction

**Figure 1:**
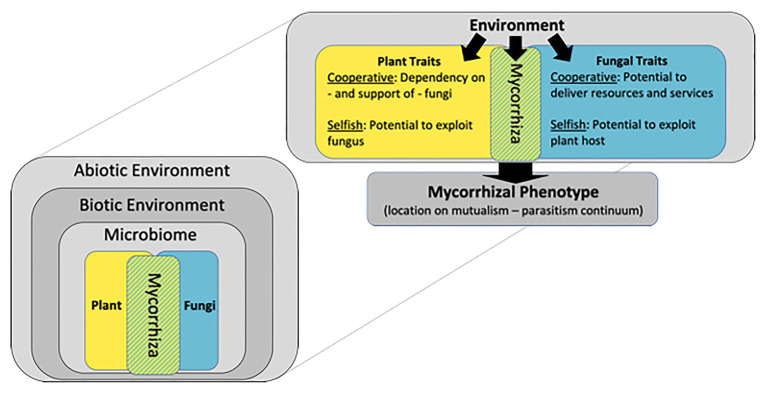
Mycorrhizae are symbiotic associations between plant roots and fungi, and their phenotype is determined by interactions among plant and fungal genotypes and the environment. A hierarchy of environmental factors determines mycorrhizal phenotype including abiotic conditions such as climate and soil properties and biotic factors such as communities of plant competitors, animal herbivores, and microbial antagonists and mutualists. Genotypes of plant and fungal partners can be characterized by cooperative traits that strengthen the mutualism and selfish traits that weaken the mutualism. Figure modified from Johnson et al. (1997).

Although the term “mycorrhiza” is often equated with a root inhabiting fungus, technically, a mycorrhiza is not a fungus, but rather the *symbiosis* between a fungus and a plant root (Frank, 1885; Trappe, 2005). Acknowledging this fact immediately expands our perspective of mycorrhizae to include not only fungi, but also their complex interactions with plant hosts. The purpose of this essay is to expand this perspective even further and envision mycorrhizae as complex adaptive systems in which matter, energy and information move through a hierarchy of interconnected components (Figure 1). Asymmetrical trading partnerships between plant hosts and arbuscular mycorrhizal (AM) fungi drive mycorrhizal systems: most plants can survive and – depending on the environment – possibly thrive in the absence of the symbiosis, while AM fungi are obligate symbionts and require a living plant host for survival. Most wild plants rely on mycorrhizae for normal nutrition, drought tolerance, and pathogen protection (Smith and Read, 2008). Although nearly all crops form AM symbioses, their value in production agriculture is debated (Ryan and Graham, 2018; Rillig et al., 2019). Envisioning mycorrhizae as constantly evolving symbiotic systems helps explain the reasons for this debate. This essay explores the hypothesis that local adaptation of mycorrhizal systems arises through multilevel selection, and that current agricultural practices uncouple critical feedbacks so that the mutualistic properties of mycorrhizas may diminish over time. An evolutionary framework can guide the design of experiments that test strategies to recouple feedbacks among plants, AM fungi and their associated microbiome so that the benefits of mycorrhizae can be harnessed in the development of sustainable agroecosystems.

## Mycorrhizal Phenotype is an Emergent Property of Mycorrhizal Systems

Phenotypes of mycorrhizal symbioses arise from the interaction between the genotypes of plant and fungal symbionts and the environment, and range along a continuum of mutualism to parasitism (Figure 1; Johnson et al., 1997). Evolution of organisms engaged in symbiotic associations is often driven by tension between cooperative traits that benefit both partners and selfish traits that benefit individuals (Bahar, 2018), and mycorrhizae are no exception. Key cooperative traits for plants involve their support of fungi in return for resources and services, while selfish plant traits involve the ability to control and manipulate AM fungi for their own benefit with minimal investment of photosynthate. In turn, cooperative traits of AM fungi involve their potential to deliver resources to host plants, and selfish traits involve their ability to overcome plant control in order to gain photosynthate without providing nutrients or beneficial services (Hammer et al., 2011; Kiers et al., 2011; Whiteside et al., 2019). In naturally evolving ecosystems these traits interact to generate a dynamic process of reciprocal adaptation between plants and fungi that is mediated by environmental heterogeneity and variation in plant and fungal traits. Natural communities are often composed of plant species that vary in the degree to which they benefit from AM symbioses (Wilson and Hartnett, 1998). There appear to be no AM fungi that are consistently beneficial to all plant hosts (Klironomos, 2003), and with the exception of mycoheterotrophic plants, there is little evidence for codependency or specificity between particular species of plants and AM fungi (Kokkoris et al., 2020a).

The scope of AM systems is much greater than individual plant-fungal partnerships, and even greater than communities of plants and AM fungi. The location of mycorrhizal phenotypes on the mutualism-parasitism continuum is an emergent property of interactions among a hierarchy of biotic and abiotic factors (Figure 1; Johnson et al., 1997). Like most organisms, plants and AM fungi host diverse microbial communities that can influence their fitness (Vandenkoornhuyse et al., 2015; Bakker et al., 2018). Currently, little is known about mycorrhizal microbiomes (Revillini et al., 2016), but there is solid evidence that undefined biotic interactions can influence plant-soil feedbacks and mediate mycorrhizal function (Hoeksema et al., 2010; De Long et al., 2019). The balance of trade between host plants and their symbiotic fungi is highly dependent on abiotic conditions and resource availability (Johnson, 2010). Temperature, precipitation, light availability, and soil chemistry can structure the environment to make AM symbioses more or less beneficial to plants. For example, symbioses between the same genotypes of plants and AM fungi have been shown to function as mutualism, commensalism, or parasitism depending on phosphorus and light availability (Johnson et al., 2015). Whether or not AM fungi form mutualistic symbioses with crop hosts depends on the biotic and abiotic conditions of the agricultural environment.

## Natural Selection, Community Assembly, and am Fungal Inoculum

Multilevel selection is the simultaneous selection of a hierarchy of groups ranging from nucleotypes within an individual AM fungal clone to whole communities of plants and microbes within an ecosystem. Evolution through natural selection occurs when heritable variation among individuals makes some phenotypes reproduce more successfully than others (Darwin, 1859). This process can be scaled up to account for evolution that results in the differential extinction and proliferation of communities (Whitham et al., 2020). Theoretical and empirical studies over the past 4 decades expand Darwinian evolution to explain how, in addition to phenotypes of individual organisms, groups of organisms can be units of selection (Bahar, 2018; Wilson, 2019; Whitham et al., 2020). This expanded paradigm of evolution is necessary for understanding local adaptation of plants as holobionts that include all organisms within their microbiome (Vandenkoornhuyse et al., 2015). It also accommodates the unusual nuclear dynamics of AM fungi in which individual clones contain thousands of independently dividing nuclei that represent genetically independent units (Kokkoris et al., 2020b). Multilevel selection blurs the distinction between genetic selection and community assembly. Multilevel selection encompasses a continuum of interactions, ranging from processes that structure the genetic composition of communities of nuclei within individual AM fungal clones to processes that structure the community composition of interacting organisms. There is evidence that mycorrhizae play a role in the adaptation of plants to their local environment in natural ecosystems (Johnson et al., 2010; Rúa et al., 2016; Remke et al., 2020), and that certain aspects of agricultural management disrupt local adaptation and may inadvertently select AM fungal communities with less mutualistic properties (Johnson et al., 1992; Verbruggen and Kiers, 2010; Verbruggen et al., 2013). Recognition of the role of multilevel selection in generating mutualistic AM phenotypes will facilitate harnessing mycorrhizae in sustainable agriculture.

The resilience and resistance of AM fungi to disturbances can be remarkable (Johnson and Wedin, 1997; Lekberg et al., 2012), and it is important to recognize that agriculture does not eliminate AM symbioses, but rather, it changes the composition of AM fungal communities to be dominated by r-selected, disturbance resistant taxa (Verbruggen and Kiers, 2010; Moora et al., 2014; Banerjee et al., 2019). Commercially produced AM fungal inoculum is generally composed of a low diversity of easily propagated r-selected AM fungi that may or may not be beneficial for nutrient uptake, drought tolerance, and pathogen protection compared to diverse communities of indigenous fungi (Berruti et al., 2016). Also, the addition of exotic AM fungal inoculum to pre-established AM fungal communities may lead to increased competition among fungi and reduced host plant productivity (Janoušková et al., 2013). These concerns have led some to suggest that the expense and risks associated with widespread application of exotic AM fungal inoculum may outweigh its potential benefits (Hart et al., 2018). Furthermore, the existence of robust communities of indigenous AM fungi in most agricultural fields implies that adding commercially produced inoculum may be like sprinkling expensive salt in the ocean.

## Flow of Matter, Energy, and Information Through Mycorrhizas

Comparing mycorrhizae in natural and agricultural ecosystems provides useful insights. Exchange of energy and matter in the form of plant photosynthate in return for fungal access to limiting soil resources is at the core of all AM symbioses. The balance of trade between plant and fungal symbionts is very different in natural and agricultural ecosystems. In drought prone, nutrient limited soils, plant hosts and AM fungi exchange valuable commodities with their symbiotic partners, but crops in highly fertilized and irrigated agricultural systems have little to gain from mycorrhizae, and if AM fungi do not provide other services such as pathogen resistance, then mycorrhizae may depress crop yield (Modjo et al., 1987; Raya-Hernández et al., 2020).

**Figure 2:**
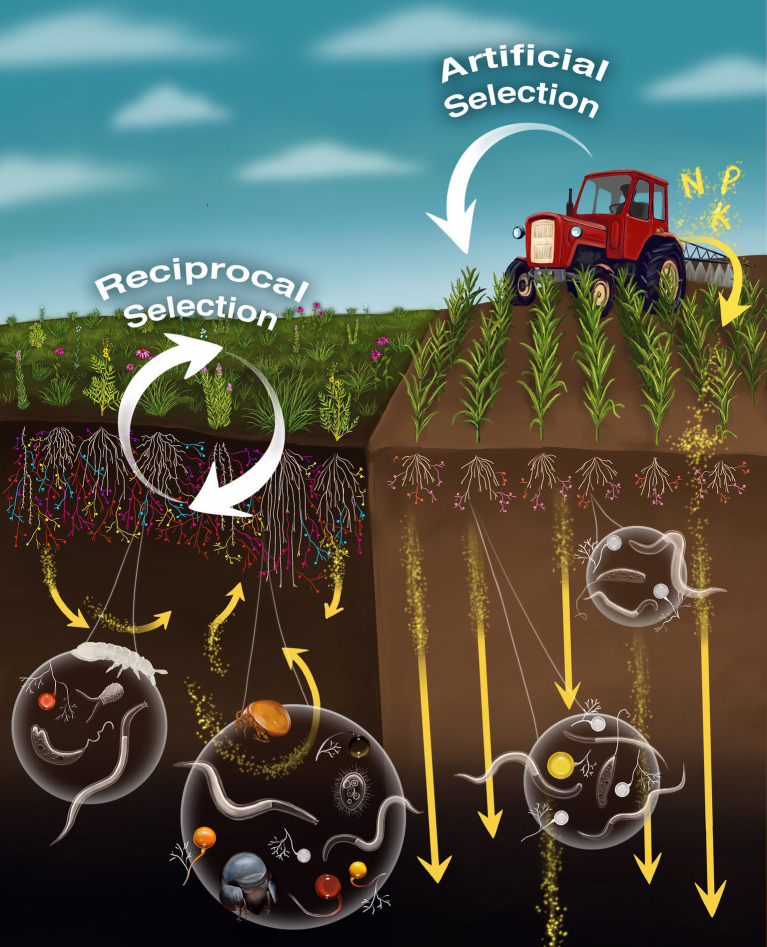
The flow of matter and energy (yellow arrows) and genetic information (white arrows) through mycorrhizae differs in natural (left) and high-input agricultural (right) ecosystems. Abundance and diversity of arbuscular mycorrhizal (AM) fungi tend to be greater in natural compared to high-input agricultural ecosystems because plants allocate more photosynthate to mycorrhizae when they are nutrient and/or water limited. Communities of soil organisms are generally more diverse in natural ecosystems. Intact networks of AM fungal hyphae and complex foodwebs help conserve nutrients in natural ecosystems, and tillage and fallow periods reduce these networks in agroecosystems so that more nutrients are lost through leaching (yellow arrows). Evolutionary feedback occurs in natural ecosystems because genotypes of plants and AM fungi are selected through reciprocal adaptation to each other and to the environment. This evolutionary feedback is not possible if farmers select plant genotypes without consideration of the indigenous AM fungi and associated microbiome. Illustration by Kara Skye Gibson.

Compared to crop monocultures, natural ecosystems have diverse plant and AM fungal communities and soil food-webs that create more complex trophic interactions, which maintain and cycle matter and energy within the system (Figure 2; Pérez-Jaramillo et al., 2016; Pärtel et al., 2017; Mariotte et al., 2018). Networks of AM hyphae have been shown to help conserve water and soil nutrients (Jia et al., 2020), and in undisturbed systems these networks remain intact year after year, throughout the seasons, in contrast to agroecosystems where regular tillage severs hyphal networks and long periods of fallow without cover-crops reduce AM fungal populations (Bowles et al., 2017). Furthermore, irrigation and fertilization may accelerate decomposition, mineralization, leaching, and volatilization processes, which when coupled with biomass removal through harvest, tend to produce open and leaky nutrient cycles in agricultural systems compared to closed and conservative nutrient cycles in natural ecosystems (Mariotte et al., 2018).

Natural and artificial selection influence the flow of genetic information through ecosystems over time. In natural ecosystems, gene frequencies and community assembly respond to local biotic and abiotic selection pressures, and the fitness of plants and fungi are reciprocally influenced by the performance of locally adapted AM symbioses (Figure 2). This reciprocity does not occur in high-input agriculture because artificial selection interrupts the feedback of information between plant and AM fungal communities. Genetic feedback is uncoupled because the farmer selects crop cultivars based on economic criteria, and the performance of AM symbioses is generally not considered. The exception to this is when traditional farmers maintain on-going selection of crops to ever changing local conditions (biotic and abiotic) by choosing the best performing phenotypes each year for the next year’s seed stock. Their choice integrates plant-soil feedback in the selection process and generates locally adapted crops and AM symbioses (Martinez and Johnson, 2010).

## Realization of Multilevel Selection in Agriculture

Mycorrhizae in natural ecosystems are complex adaptive systems with feedback between communities of plants and soil organisms, but high-input agricultural management has inadvertently removed these feedbacks. Mycorrhizae in high-input agriculture are not complex adaptive systems because: (1) fertilization, irrigation, and pesticides remove resource limitation and stress, (2) AM fungi are not selected by plants to enhance cooperative traits like nutrient uptake or pathogen resistance, and instead, AM fungi are selected for selfish traits that enhance their own survival in an environment that does not favor mycotrophy, and (3) crop traits are completely controlled by the farmer’s choice of seed, not through evolutionary feedbacks from the environment and soil biota.

A better understanding of how multilevel selection controls mycorrhizal functioning in natural ecosystems may provide insights into ways to effectively harness mycorrhizal benefits in modern agroecosystems. The sustainable agriculture movement aims to enhance environmental quality, maintain soil fertility, reduce erosion, and sustain the economic viability of farm operations (USDA, 2007). Managing for mycorrhizae may help achieve these goals. Management practices that maximize mycorrhizal benefits include planting cover crops and minimizing use of tillage and fertilizers (Bowles et al., 2017; Banerjee et al., 2019). Locally adapted communities of AM fungi and other soil microbes may help protect crops from site-specific stresses (Johnson et al., 2010; Remke et al., 2020); and maintaining intact networks of AM fungal mycelium may increase soil organic matter and improve nutrient retention, soil stability, and drought tolerance (Zhu and Miller, 2003; Gosling et al., 2006; Jia et al., 2020).

### Darwinian Agriculture in the 21st Century

The traditional narrow focus on individuals as the only unit of selection (Williams, 1966) has been shaken by the discovery that individual organisms are not individuals, but really complex communities of bacteria, archaea, viruses, fungi, and other organisms. Furthermore, horizontal gene transfer allows adaptive genes to be readily exchanged among unrelated organisms. The asexual fungi that form AM symbioses carry variable nuclei in their hyphae and spores such that nucleotypes within the same fungal clone may be differentially selected by fine-scale heterogeneity in environmental conditions (Rosendahl, 2008; Kokkoris et al., 2020b). So, what is the unit of selection? Does selection occur at a highly localized scale with certain genes increasing in abundance in the specific locations where they are the most adaptive? Do organisms actively select teams of nucleotypes of microorganisms to help them adapt to stresses in their local environment? Darwin developed the theory of evolution through natural selection without a knowledge of genetics or microbiomes. Julian Huxley merged genetics with Darwin’s theory in his 1942 book *Evolution: The Modern Synthesis*. It is time to update the modern synthesis to include microbiomes.

### How Can Agricultural Management Maximize Beneficial Mycorrhizae?

Mechanistic insights informing the development of sustainable agricultural systems may be gained through systematic testing of hypotheses related to the mycorrhizal phenotype model (Figure 1). The focus should be on generating conditions that favor cooperative traits and minimize selfish traits in both plant and fungal partners. In addition to judicious management of fertilizers, crop breeders should identify particular physiological and morphological traits that influence the degree to which crops utilize AM symbioses. This would allow farmers to select crop cultivars that vary in their level of mycotrophy to account for the nutrient and water availability in their fields. When soil resources are in ample supply, it may be best to grow cultivars that minimize AM colonization, but when nutrients and water are limited, farmers should select cultivars that leverage symbioses with indigenous AM fungi that are best adapted to the local stresses. Prior research suggests the following list of hypothesized principles for *in situ* management of mycorrhizas in agroecosystems:

1. To reduce fertilizer inputs, develop mycotrophic crop varieties that substitute symbiotic uptake of nutrients for fertilizer supplements.
2. Maintain soil P at levels that encourage selection of mutualistic mycorrhizae which optimize trading of minerals for photosynthate but do not limit crop yield.
3. Provide sufficient N to prevent N-competition between plants and AM fungi.
4. Maintain soil water availability so that mycorrhizal enhancement of drought tolerance can be manifested, but not so low that AM fungi are chronically water limited.
5. Reduce tillage to maintain the fine-scale spatial structure necessary for intact hyphal networks to evolve over time through multilevel selection.
6. Maintain host plant continuity through planting mycotrophic cover crops or perennial crop varieties.
7. Add spatial or temporal diversity through crop rotation, intercropping or other polyculture practices.

Field-based studies in many different environments are necessary to sufficiently test whether or not these hypothesized principles maximize mycorrhizal mutualism and to develop strategies to enhance their value for crop production and other ecosystem services.

Managing biological soil fertility in general and mycorrhizal symbioses in particular is environmentally sustainable, but it may not be economically sustainable under current market conditions (Gowdy and Baveye, 2019). Many conventional agricultural practices such as application of inorganic fertilizers, irrigation, and selection of non-mycotrophic cultivars make the management of mycorrhizal symbioses superfluous for optimizing crop performance (Ryan and Graham, 2018). These high-input agricultural practices are perpetuated by economic forces that do not account for externalities such as greenhouse gas emissions, groundwater contamination, and topsoil erosion and degradation. Managing mycorrhizae may only make economic sense if all of the environmental costs of agriculture are monetized and accounted for in farmers’ profits. National and international policies are necessary to combat climate change and protect common pool resources such as topsoil, ground water, and mineral P stocks. Awareness of the potential benefits of locally adapted mycorrhizal symbioses for their many ecosystem services is an important step toward the design and implementation of truly sustainable agriculture.

## Data Availability Statement

The original contributions presented in the study are included in the article/supplementary material; further inquiries can be directed to the corresponding author/s.

## Author Contributions

NJ drafted the initial version of the manuscript. KG contributed critical ideas and created Figure 2. All authors contributed to the article and approved the submitted version.

### Conflict of Interest

The authors declare that the research was conducted in the absence of any commercial or financial relationships that could be construed as a potential conflict of interest.

## References

[ref1] Bahar S. (2018). The essential tension: Competition, cooperation and multilevel selection in evolution. 1st Edn. Netherlands: Springer, 377.

[ref2] Bakker P. A. H. M. Pieterse C. M. J. de Jonge R. Berendsen R. L. (2018). The soil-borne legacy. Cell 172, 1178–1180. 10.1016/j.cell.2018.02.024, PMID: 29522740

[ref3] Banerjee S. Walder F. Büchi L. Meyer M. Held A. Y. Gattinger A.. (2019). Agricultural intensification reduces microbial network complexity and the abundance of keystone taxa in roots. ISME J. 13, 1722–1736. 10.1038/s41396-019-0383-2, PMID: 30850707 PMC6591126

[ref4] Berruti A. Lumini E. Balestrini R. Bianciotto V. (2016). Arbuscular mycorrhizal fungi as natural biofertilizers: let’s benefit from past successes. Front. Microbiol. 6:1559. 10.3389/fmicb.2015.01559, PMID: 26834714 PMC4717633

[ref5] Bowles T. M. Jackson L. E. Loeher M. Cavagnaro T. R. (2017). Ecological intensification and arbuscular mycorrhizas: a meta-analysis of tillage and cover crop effects. J. Appl. Ecol. 54, 1785–1793. 10.1111/1365-2664.12815

[ref6] Darwin C. (1859). The origin of species: By means of natural selection, or the preservation of favoured races in the struggle for life. 1st Edn. New York: D. Appleton and Company.

[ref7] De Long J. R. Fry E. L. Veen G. F. Kardol P. (2019). Why are plant–soil feedbacks so unpredictable, and what to do about it? Funct. Ecol. 33, 118–128. 10.1111/1365-2435.13232

[ref8] Frank A. B. (1885). Ueber die auf Wurzelsymbiose beruhende Ernährung gewisser Bäume durch unterirdische Pilze. Ber Dtsch Bot Ges 3, 128–145 [English translation (2005), *Mycorrhiza* 15, 267-275]. 10.1007/s00572-004-0329-y

[ref9] Gosling P. Hodge A. Goodlass G. Bending G. D. (2006). Arbuscular mycorrhizal fungi and organic farming. Agric. Ecosyst. Environ. 113, 17–35. 10.1016/j.agee.2005.09.009

[ref10] Gowdy J. Baveye P. (2019). “An evolutionary perspective on industrial and sustainable agriculture” in Agroecosystem diversity: Reconciling contemporary agriculture and environmental quality. ed. Lemaire G. (Amsterdam: Elsevier), 433–435.

[ref11] Hammer E. C. Pallon J. Wallander H. Olsson P. A. (2011). Tit for tat? A mycorrhizal fungus accumulates phosphorus under low plant carbon availability. FEMS Microbiol. Ecol. 76, 236–244. 10.1111/j.1574-6941.2011.01043.x, PMID: 21223336

[ref12] Hart M. M. Antunes P. M. Chaudhary V. B. Abbott L. K. (2018). Fungal inoculants in the field: is the reward greater than the risk? Funct. Ecol. 32, 126–135. 10.1111/1365-2435.12976

[ref13] Hoeksema J. D. Chaudhary V. B. Gehring C. A. Johnson N. C. Karst J. Koide R. T.. (2010). A meta-analysis of context-dependency in plant response to inoculation with mycorrhizal fungi. Ecol. Lett. 13, 394–407. 10.1111/j.1461-0248.2009.01430.x, PMID: 20100237

[ref14] Janoušková M. Krak K. Wagg C. Štorchová H. Caklová P. Vosátka M. (2013). Effects of inoculum additions in the presence of a preestablished arbuscular mycorrhizal fungal community. Appl. Environ. Microbiol. 79, 6507–6515. 10.1128/AEM.02135-13, PMID: 23956395 PMC3811198

[ref15] Jia Y. van der Heijden M. G. A. Wagg C. Feng G. Walder F. (2020). Symbiotic soil fungi enhance resistance and resilience of an experimental grassland to drought and nitrogen deposition. J. Ecol. 10.1111/1365-2745.13521

[ref16] Johnson N. C. (2010). Resource stoichiometry elucidates the structure and function of arbuscular mycorrhizas across scales. New Phytol. 185, 631–647. 10.1111/j.1469-8137.2009.03110.x, PMID: 19968797

[ref17] Johnson N. C. Copeland P. J. Crookston R. K. Pfleger F. L. (1992). Mycorrhizae: possible explanation for yield decline with continuous corn and soybean. Agron. J. 84, 387–390. 10.2134/agronj1992.00021962008400030007x

[ref18] Johnson N. C. Graham J. H. Smith F. A. (1997). Functioning of mycorrhizal associations along the mutualism-parasitism continuum. New Phytol. 135, 575–585. 10.1046/j.1469-8137.1997.00729.x

[ref19] Johnson N. C. Wedin D. A. (1997). Soil carbon, nutrients, and mycorrhizae during conversion of dry tropical forest to grassland. Ecol. Appl. 7, 171–182. 10.1890/1051-0761(1997)007[0171:SCNAMD]2.0.CO;2

[ref20] Johnson N. C. Wilson G. W. T. Bowker M. A. Wilson J. A. Miller R. M. (2010). Resource limitation is a driver of local adaptation in mycorrhizal symbioses. Proc. Natl. Acad. Sci. U. S. A. 107, 2093–2098. 10.1073/pnas.0906710107, PMID: 20133855 PMC2836645

[ref21] Johnson N. C. Wilson G. W. T. Wilson J. A. Miller R. M. Bowker M. A. (2015). Mycorrhizal phenotypes and the law of the minimum. New Phytol. 205, 1473–1484. 10.1111/nph.13172, PMID: 25417818

[ref22] Kiers E. T. Duhamel M. Beesetty Y. Mensah J. A. Franken O. Verbruggen E.. (2011). Reciprocal rewards stabilize cooperation in the mycorrhizal symbiosis. Science 333, 880–882. 10.1126/science.1208473, PMID: 21836016

[ref23] Klironomos J. N. (2003). Variation in plant response to native and exotic arbuscular mycorrhizal fungi. Ecology 84, 2292–2301. 10.1890/02-0413

[ref24] Kokkoris V. Lekberg Y. Antunes P. M. Fahey C. Fordyce J. A. Kivlin S. N. (2020a). Codependency between plant and arbuscular mycorrhizal fungal communities: what is the evidence? New Phytol. 228, 828–838. 10.1111/nph.16676 32452032

[ref25] Kokkoris V. Stefani F. Dalpé Y. Dettman J. Corradi N. (2020b). Nuclear dynamics in the arbuscular mycorrhizal fungi. Trends Plant Sci. 25, 765–778. 10.1016/j.tplants.2020.05.002 32534868

[ref26] Lekberg Y. Schnoor T. Kjøller R. Gibbons S. M. Hansen L. H. Al-Soud W. A. (2012). 454-sequencing reveals stochastic local reassembly and high disturbance tolerance within arbuscular mycorrhizal fungal communities. J. Ecol. 100, 151–160. 10.1111/j.1365-2745.2011.01894.x

[ref27] Mariotte P. Mehrabi Z. Bezemer T. M. De Deyn G. B. Kulmatiski A. Drigo B.. (2018). Plant–soil feedback: bridging natural and agricultural sciences. Trends Ecol. Evol. 33, 129–142. 10.1016/j.tree.2017.11.005, PMID: 29241940

[ref28] Martinez T. N. Johnson N. C. (2010). Agricultural management influences propagule densities and functioning of arbuscular mycorrhizas in low- and high-input agroecosystems in arid environments. Appl. Soil Ecol. 46, 300–306. 10.1016/j.apsoil.2010.07.001

[ref29] Modjo H. S. Hendrix J. W. Nesmith W. C. (1987). Mycorrhizal fungi in relation to control of tobacco stunt disease with soil fumigants. Soil Biol. Biochem. 19, 289–295. 10.1016/0038-0717(87)90011-3

[ref30] Moora M. Davison J. Öpik M. Metsis M. Saks Ü. Jairus T.. (2014). Anthropogenic land use shapes the composition and phylogenetic structure of soil arbuscular mycorrhizal fungal communities. FEMS Microbiol. Ecol. 90, 609–621. 10.1111/1574-6941.12420, PMID: 25187481

[ref31] Pärtel M. Öpik M. Moora M. Tedersoo L. Szava-Kovats R. Rosendahl S.. (2017). Historical biome distribution and recent human distrubance shape the diversity of arbuscular mycorrhizal fungi. New Phytol. 216, 227–238. 10.1111/nph.14695, PMID: 28722181

[ref32] Pérez-Jaramillo J. E. Mendes R. Raaijmakers J. M. (2016). Impact of plant domestication on rhizosphere microbiome assembly and functions. Plant Mol. Biol. 90, 635–644. 10.1007/s11103-015-0337-7, PMID: 26085172 PMC4819786

[ref33] Raya-Hernández A. I. Jaramillo-López P. F. López-Carmona D. A. Díaz T. Carrera-Valtierra J. A. Larsen J. (2020). Field evidence for maize-mycorrhiza interactions in agroecosystems with low and high P soils under mineral and organic fertilization. Appl. Soil Ecol. 149:103511. 10.1016/j.apsoil.2020.103511

[ref34] Remke M. Johnson N. C. Wright J. Williamson M. Bowker M. (2020). Sympatric pairings of dryland grass populations, mycorrhizal fungi, and associated soil biota enhane mutualism and ameliorate drought stress. J. Ecol. 10.1111/1365-2745.13546

[ref35] Revillini D. Gehring C. A. Johnson N. C. (2016). The role of locally adapted mycorrhizas and rhizobacteria in plant–soil feedback systems. Funct. Ecol. 30, 1086–1098. 10.1111/1365-2435.12668

[ref36] Rillig M. C. Aguilar-Trigueros C. A. Camenzind T. Cavagnaro T. R. Degrune F. Hohmann P.. (2019). Why farmers should manage the arbuscular mycorrhizal symbiosis. New Phytol. 222, 1171–1175. 10.1111/nph.15602, PMID: 30657593

[ref37] Rosendahl S. (2008). Communities, populations and individuals of arbuscular mycorrhizal fungi. New Phytol. 178, 253–266. 10.1111/j.1469-8137.2008.02378.x, PMID: 18248587

[ref38] Rúa M. A. Antoninka A. Antunes P. M. Chaudhary V. B. Gehring C. Lamit L. J. (2016). Home-field advantage? Evidence of local adaptation among plants, soil, and arbuscular mycorrhizal fungi through meta-analysis. BMC Evol. Biol. 16:122. 10.1186/s12862-016-0698-9 27287440 PMC4902977

[ref39] Ryan M. H. Graham J. H. (2018). Little evidence that farmers should consider abundance or diversity of arbuscular mycorrhizal fungi when managing crops. New Phytol. 220, 1092–1107. 10.1111/nph.15308, PMID: 29987890

[ref40] Smith S. E. Read D. J. (2008). Mycorrhizal symbiosis. 3rd Edn. Cambridge, Massachusetts: Academic Press.

[ref41] Trappe J. M. (2005). A.B. Frank and mycorrhizae: the challenge to evolutionary and ecologic theory. Mycorrhiza 15, 277–281. 10.1007/s00572-004-0330-5, PMID: 15503185

[ref42] USDA (2007). National Agriculture Library. Special Reference Briefs Series no. SRB 99-02 Update SRB 94-05 Sustainable Agriculture: Definitions and Terms. Available at: https://www.nal.usda.gov/afsic/sustainable-agriculture-definitions-and-terms (Accessed December 12, 2020).

[ref43] Vandenkoornhuyse P. Quaiser A. Duhamel M. Le Van A. Dufresne A. (2015). The importance of the microbiome of the plant holobiont. New Phytol. 206, 1196–1206. 10.1111/nph.13312, PMID: 25655016

[ref44] Verbruggen E. Kiers E. T. (2010). Evolutionary ecology of mycorrhizal functional diversity in agricultural systems. Evol. Appl. 3, 547–560. 10.1111/j.1752-4571.2010.00145.x, PMID: 25567946 PMC3352509

[ref45] Verbruggen E. van der Heijden M. G. A. Rillig M. C. Kiers E. T. (2013). Mycorrhizal fungal establishment in agricultural soils: factors determining inoculation success. New Phytol. 197, 1104–1109. 10.1111/j.1469-8137.2012.04348.x, PMID: 23495389

[ref46] Whiteside M. D. Werner G. D. A. Caldas V. E. A. van’t Padje A. Dupin S. E. Elbers B.. (2019). Mycorrhizal fungi respond to resource inequality by moving phosphorus from rich to poor patches across networks. Curr. Biol. 29, 2043.e8–2050.e8. 10.1016/j.cub.2019.04.061, PMID: 31178314 PMC6584331

[ref47] Whitham T. G. Allan G. J. Cooper H. F. Shuster S. M. (2020). Intraspecific genetic variation and species interactions contribute to community evolution. Annu. Rev. Ecol. Evol. Syst. 51, 587–612. 10.1146/annurev-ecolsys-011720-123655

[ref48] Williams G. C. (1966). Adaptation and natural selection. Princeton, New Jersey: Princeton University Press.

[ref49] Wilson D. S. (2019). This view of life: Completing the Darwinian revolution. 1st Edn. New York: Pantheon Books, 288.

[ref50] Wilson G. W. T. Hartnett D. C. (1998). Interspecific variation in plant responses to mycorrhizal colonization in tallgrass prairie. Am. J. Bot. 85, 1732–1738. 10.2307/2446507, PMID: 21680333

[ref51] Zhu Y. G. Miller R. M. (2003). Carbon cycling by arbuscular mycorrhizal fungi in soil-plant systems. Trends Plant Sci. 8, 407–409. 10.1016/S1360-1385(03)00184-5, PMID: 13678905

